# Assessing congruence of opportunistic records and systematic surveys for predicting Hispaniolan mammal species distributions

**DOI:** 10.1002/ece3.6258

**Published:** 2020-05-23

**Authors:** Samuel T. Turvey, Rosalind J. Kennerley, Michael A. Hudson, Jose M. Nuñez‐Miño, Richard P. Young

**Affiliations:** ^1^ Institute of Zoology Zoological Society of London Regent’s Park, London UK; ^2^ Durrell Wildlife Conservation Trust Trinity, Jersey Channel Islands

**Keywords:** Dominican Republic, historical records, hutia, maxent, solenodon, species distribution model

## Abstract

Comparative assessment of the relative information content of different independent spatial data types is necessary to evaluate whether they provide congruent biogeographic signals for predicting species ranges. Opportunistic occurrence records and systematically collected survey data are available from the Dominican Republic for Hispaniola’s surviving endemic non‐volant mammals, the Hispaniolan solenodon (*Solenodon paradoxus*) and Hispaniolan hutia (*Plagiodontia aedium*); opportunistic records (archaeological, historical and recent) exist from across the entire country, and systematic survey data have been collected from seven protected areas. Species distribution models were developed in maxent for solenodons and hutias using both data types, with species habitat suitability and potential country‐level distribution predicted using seven biotic and abiotic environmental variables. Three different models were produced and compared for each species: (a) opportunistic model, with starting model incorporating abiotic‐only predictors; (b) total survey model, with starting model incorporating biotic and abiotic predictors; and (c) reduced survey model, with starting model incorporating abiotic‐only predictors to allow further comparison with the opportunistic model. All models predict suitable environmental conditions for both solenodons and hutias across a broadly congruent, relatively large area of the Dominican Republic, providing a spatial baseline of conservation‐priority landscapes that might support native mammals. Correlation between total and reduced survey models is high for both species, indicating the substantial explanatory power of abiotic variables for predicting Hispaniolan mammal distributions. However, correlation between survey models and opportunistic models is only moderately positive. Species distribution models derived from different data types can provide different predictions about habitat suitability and conservation‐priority landscapes for threatened species, likely reflecting incompleteness and bias in spatial sampling associated with both data types. Models derived using both opportunistic and systematic data must therefore be applied critically and cautiously.

## Graphical abstract

Comparative assessment of the relative information content of opportunistic historical records and survey data was conducted for Hispaniolan solenodon and Hispaniolan hutia, to evaluate whether these different data types provide congruent predictions of species distributions. Correlation between survey models and opportunistic models is only moderately positive, likely reflecting incompleteness and bias in spatial sampling, and indicating that models derived using both data types must be applied critically and cautiously.

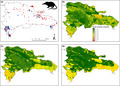

## 1 INTRODUCTION

Scientific data are crucial to inform decision‐making and improve the efficiency of management interventions in evidence‐based conservation (Sutherland, Pullin, Dolman, & Knight, 2004). However, although methodologies for evaluating conservation evidence have been defined and standardized (Pullin & Stewart, 2006), multiple conservation‐relevant data sources can be available to decision‐makers, which might contain different types of information and therefore potentially provide different insights for management (Adams & Sandbrook, 2013; Bower et al., 2018). Systematically collected datasets on key conservation‐relevant parameters are also often unavailable for threatened species that require urgent targeted mitigations, such that limited and biased opportunistically collected “anecdotal” data might constitute the only baseline available to guide management decisions (Stewart, Coles, & Pullin, 2005; Thompson, 2013). In particular, spatial data for reconstructing geographic distributions are often unevenly sampled for threatened species, with systematically derived data available only for the subset of sites that have been surveyed; this can hinder assessment of ecological requirements, threats, and landscape‐level conservation prioritization (Boakes et al., 2010; Boitani et al., 2011; Guisan et al., 2013), especially for species that occur across large geographic areas (Marris, 2007).

A common approach to compensate for limited availability of spatial occurrence records is the use of species distribution models (SDMs). These models predict distribution in environmental space from distribution in geographic space, by identifying statistical relationships between species occurrence records and sets of environmental variables in order to identify locations where species are expected to occur (Franklin, 2009; Guisan et al., 2013). SDMs have been used to generate spatially explicit predictions of environmental suitability and to forecast and hindcast species ranges and range changes using various predictive environmental scenarios, and considerable attention has been paid to factors that might affect the accuracy of range prediction, including data quantity, quality and representativeness, and randomness of sampling (Feeley & Silman, 2011; Fei & Yu, 2016; Fithian, Elith, Hastie, & Keith, 2015). In practice, however, SDMs are frequently forced to rely on historical occurrence records (e.g., museum records) that have been collected opportunistically rather than systematically, are of varying spatial resolution, and/or include bias in spatial search effort (Boakes et al., 2010; Loiselle et al., 2003; Lütolf, Kienast, & Guisan, 2006; Tingley & Beissinger, 2009). As such incomplete and biased datasets often constitute the only information available to determine potential geographic distributions for threatened species, it is necessary to assess the information content of such data for conservation and evaluate whether they provide a meaningful biogeographic signal.

**FIGURE 1:**
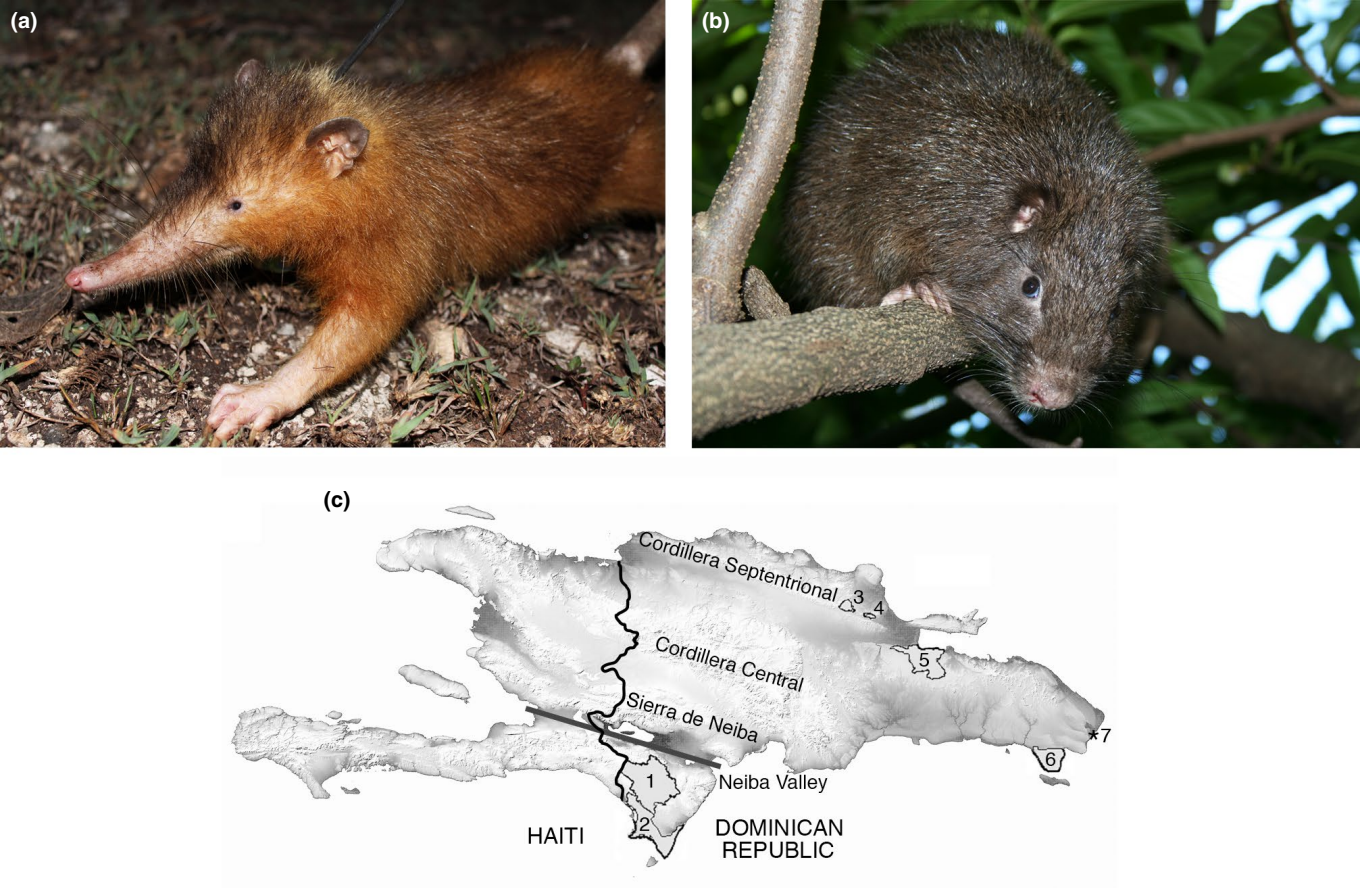
(a) Hispaniolan solenodon (*Solenodon paradoxus*). Photograph courtesy of Rocio Pozo Rodríguez. (b) Hispaniolan hutia (*Plagiodontia aedium*). Photograph copyright José Nuñez‐Miño/The Last Survivors project. (c) Map of the Dominican Republic, showing localities mentioned in the text: 1, Sierra de Bahoruco National Park; 2, Jaragua National Park; 3, Loma Quita Espuela Scientific Reserve; 4, Loma Guaconejo Scientific Reserve; 5, Los Haitises National Park; 6, Del Este National Park; 7, Punta Cana Ecological Reserve

The insular Caribbean experienced a severe postglacial mammal extinction event and contains few surviving native land mammal species, most of which are threatened with extinction (Cooke, Dávalos, Mychajliw, Turvey, & Upham, 2017; Turvey, Kennerley, Nuñez‐Miño, & Young, 2017). Hispaniola, the second‐largest Caribbean island (divided politically into the Dominican Republic and Haiti), retains only two nonvolant endemic land mammals: the Hispaniolan solenodon (*Solenodon paradoxus*), a large eulipotyphlan insectivore, and the Hispaniolan hutia (*Plagiodontia aedium*), a large capromyid rodent (Figure 1). Both species are listed as Endangered by IUCN (2018) and are recognized as global conservation priorities based on evolutionary distinctiveness (Collen et al., 2011). The biology and ecology of Hispaniolan mammals are poorly understood, due to their apparent rarity and secretive nocturnal behavior. Both species are largely extirpated from Haiti, surviving only as tiny remnant populations in the south of the country (Turvey et al., 2014; Woods, 1981; Woods & Ottenwalder, 1992), but their distribution across the Dominican Republic is unclear. They have always been considered very rare and in danger of extinction in the Dominican Republic, if not already extinct (Allen, 1942; Fisher & Blomberg, 2011; Verrill, 1907), but visiting naturalists have reported opportunistic observations of both species widely across the country over the past century. Surveys periodically conducted in the 1970s and 1980s documented the presence of both species in several landscapes, but these studies typically failed to report survey effort, field methods, or even many precise localities, or to provide analyses or quantitative results (Ottenwalder, 1991, 1999; Sullivan, 1983). The only large‐scale systematic survey of the ecology and distribution of native land mammals in the Dominican Republic was conducted across seven protected areas in 2010–2012 (Kennerley, Nicoll, Young, et al., 2019).

Assessing the country‐wide distribution of Hispaniola's endemic land mammals is an important conservation research priority needed to inform national‐level management and spatial allocation of resources for these protected species (Martínez et al., 2013), understand the likely impact of potential threats, and reassess global threat status (Turvey et al., 2017). Hispaniola is geologically and environmentally heterogeneous, and contains a complex diversity of ecosystems across lowland and montane landscapes (Ottenwalder, 1999; Figure 1), making it hard to predict spatial patterns of endemic mammal occurrence and distribution in the absence of robust data. Species distribution modeling to predict future responses to climate change has recently been conducted for Hispaniolan solenodons, using historical and fossil data and recent local‐scale encounter records (Gibson, Mychajliw, Leon, Rupp, & Hadly, 2019). However, nonsystematically collected data (including opportunistic records by visiting naturalists, older qualitatively reported survey records, and other data such as Holocene archaeological records) and recent systematic survey data constitute two independent sets of data available to understand spatial distributions of both of Hispaniola's native nonvolant land mammals, providing a useful opportunity to assess the relative information content and predictive ability of the two main categories of spatial data that are typically available to reconstruct species ranges. We therefore developed separate comparative SDMs for both Hispaniolan solenodons and Hispaniolan hutias using data from opportunistic historical records and systematic surveys, respectively, to determine the congruence of spatially explicit range predictions based on different data types. Our findings provide a new baseline for understanding the spatial conservation requirements and status of Hispaniolan mammals, and have wider implications for assessing the potential representativeness of nonsystematic data for inferring geographic distributions and understanding spatial ecology in other poorly known species.

## 2 METHODS

### 2.1 Presence records

**FIGURE 2:**
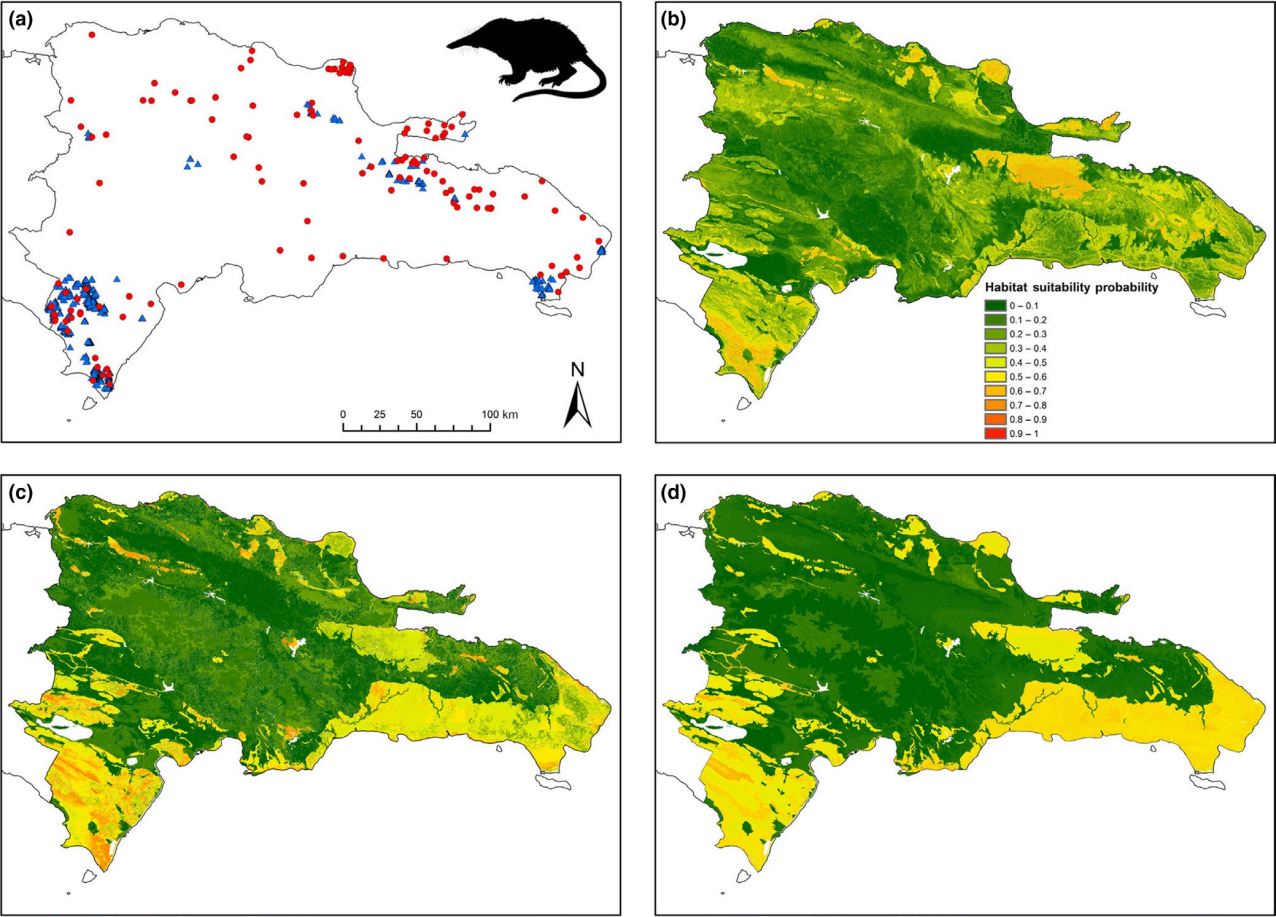
(a) Occurrence records for Hispaniolan solenodon (*Solenodon paradoxus*) across the Dominican Republic: red circles, opportunistic locality records (*n* = 135); blue triangles, systematic survey records (*n* = 867). (b–d) Species distribution models for the Dominican Republic based on solenodon occurrence data: (b) opportunistic model; (c) total survey model; (d) reduced survey model

**FIGURE 3:**
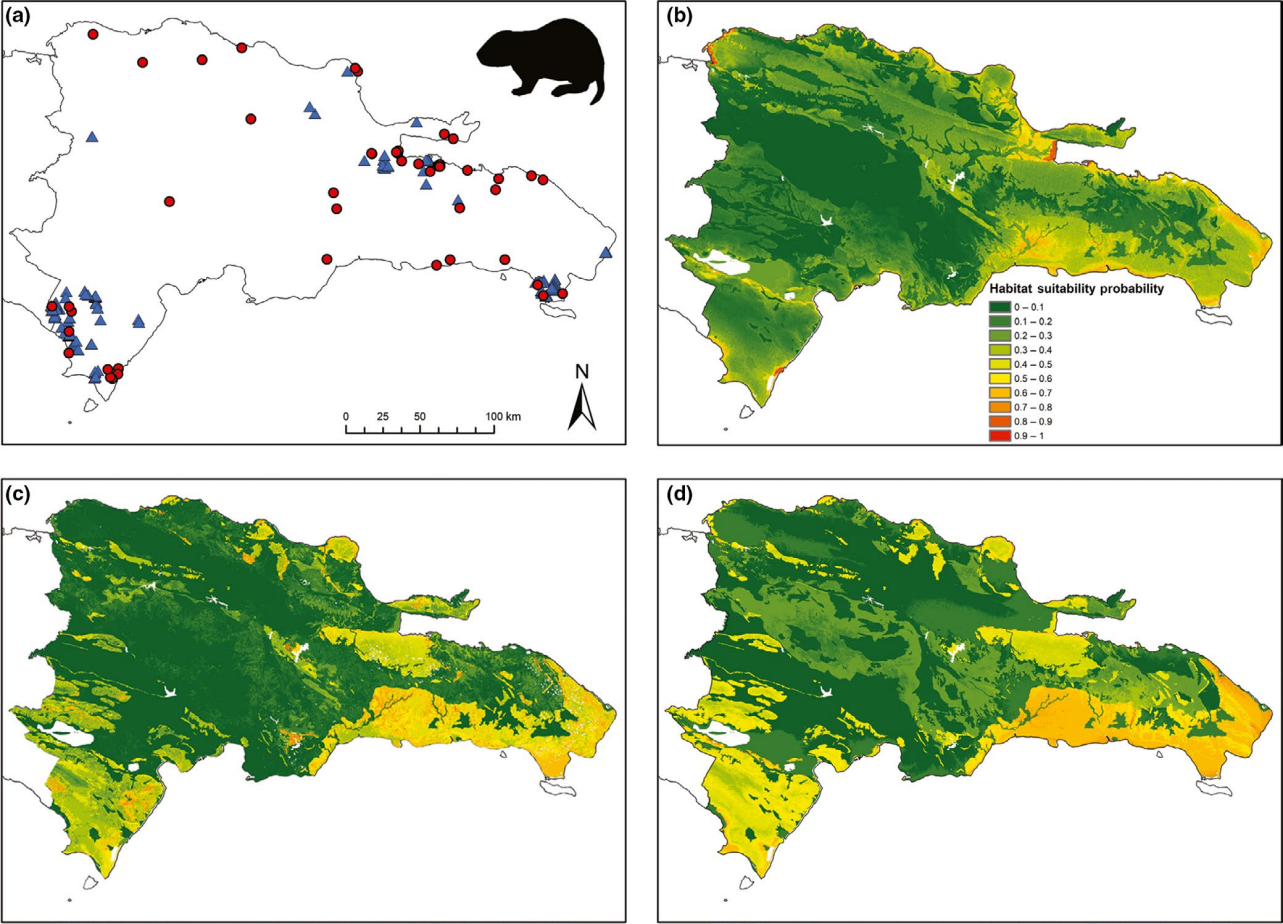
(a) Occurrence records for Hispaniolan hutia (*Plagiodontia aedium*) across the Dominican Republic: red circles, opportunistic locality records (*n* = 48); blue triangles, systematic survey records (*n* = 240). (b–d) Species distribution models for the Dominican Republic based on hutia occurrence data: (b) opportunistic model; (c) total survey model; (d) reduced survey model

We collected opportunistic locality records for solenodons (*n* = 135) and hutias (*n* = 48) in the Dominican Republic from the published literature, museum accession records, and personal communication with other field biologists (Figures 2 and 3; https://doi.org/10.5522/04/11993388.v1). We excluded additional records that reported nonspecific or vaguely described localities. Opportunistic records dated from the late Holocene pre‐Columbian archaeological period to the late 20th century; we excluded Pleistocene or undated Late Quaternary records because they may represent premodern environmental conditions. Historical and archaeological records identified as the extant species *Plagiodontia hylaeum* or the extinct species *P. ipnaeum* and *P. caletensis* were included within *P. aedium*, as these taxa are now recognized as synonyms (Hansford et al., 2012). We assigned a geographic coordinate (latitude–longitude) for all locality points by georeferencing them in Google Earth (https://earth.google.com/web).

We collected systematic survey data at 289 randomized survey points within seven protected areas, representing both national parks (NPs) and privately owned protected areas distributed across the Dominican Republic, and covering a wide range of habitats, vegetation types, and topographic and climatic variables (Figure 1, Table 1). We determined the species presence using diagnostic indirect signs for both species (solenodon foraging “nose pokes”; evidence of hutia feeding/gnawing on fruit, bark, and leaves; feces of both species; Mohr, 1936–1938; Ottenwalder, 1999). Point selection within most protected areas was random; we stratified Sierra de Bahoruco NP into 400‐m elevational bands (~20 points per stratum) to ensure all habitat types were surveyed, and stratified survey effort by vegetation type in Jaragua and Del Este NPs (Jaragua categories: low/no vegetation cover, dry scrub, dry forest, semihumid broadleaf forest, mangrove; Del Este categories: mangrove, semihumid broadleaf forest, broadleaf scrub). Full details of sampling methodology are given in Kennerley, Nicoll, Young, et al. (2019). Systematic surveys detected 867 presence records for solenodons and 240 presence records for hutias, including data from randomized survey points and additional fieldwork records (signs found when traveling between survey points; local informant observations of live or dead animals) (https://doi.org/10.5522/04/11993424.v1).

**TABLE 1:** Summary data for seven protected areas in the Dominican Republic where systematic survey data were collected for endemic land mammals

Protected area	Area (km^2^)	Coordinates	Elevation (masl)	Habitat types	Survey points	Fieldwork dates
Sierra de Bahoruco NP	1,125	18°10’*N*, 71°31’W	300–2367	Dry and wet broadleaf forest, pine forest	168	05/03/10 to 20/04/11
Jaragua NP	1,654	17°49’*N*, 71°32’W	0–331	Dry forest, mangroves, coastal wetlands	22	19/07/10 to 14/01/11
Loma Quita Espuela Scientific Reserve	92	19°23’*N*, 70°08’W	100–985	Subtropical moist forest, cloud forest, rainforest, riparian forest, wetlands	19	11/08/11 to 19/12/11
Loma Guaconejo Scientific Reserve	23	19°19’*N*, 69°59’W	0–606	Broadleaf forest, broadleaf scrub, pasturelands	19	05/06/12 to 11/06/12
Los Haitises NP	634	19°01’*N*, 69°37’W	0–287	Tropical moist forest, karst forest, mangroves, wetlands, coastal forest	40	13/08/11 to 23/06/12
Del Este NP	428	18°16’*N*, 68°42’W	0–60	Broadleaf forest, karst forest, scrub, savanna, wetlands	16	06/07/10 to 17/06/11
Punta Cana Ecological Reserve	11	18°32’*N*, 68°22’W	0–15	Coastal scrub, older secondary‐growth dry forest	5	10/08/10 to 11/08/10

### 2.2 Environmental data

We predicted Hispaniolan mammal habitat suitability and potential distribution using five continuous environmental variables (elevation, slope, aspect, percentage forest cover, and distance to nearest road) and two categorical environmental variables (geology type and land cover type). We calculated elevation using a 30‐m resolution ASTER Global Digital Elevation Model (METI & NASA, 2011), from which separate layers were calculated for slope, aspect (cosine), and aspect (sine). We calculated forest cover using 30‐m resolution tree cover data from 2000 (Hansen et al., 2013), which defines canopy closure for all vegetation >5 m in height. Detailed data on human settlements and population density across the Dominican Republic are not available, so we used distance to nearest road as a proxy measure of degree of isolation from human activity, calculated from road data obtained from DIVA‐GIS (Hijmans et al., 2004) and incorporating topographic variation and Euclidean distance (Blake et al., 2007). We obtained geological data from Dirección General de Minería (2010), combining similar rock types into broader grouped geological categories for analysis (e.g., basalt, fluvial deposit, limestone), to prevent specific categories from being underrepresented in models, and resulting in a final set of 35 categories. We obtained land cover data for 2004 (representing the most recent publically available data) from the Dominican Republic Ministry of Environment (cf. Sangermano et al., 2015); we again combined similar habitat types into broader grouped categories for analysis, resulting in a final set of 45 categories. Additional bioclimatic variables (e.g., from www.worldclim.org) could not be included because these are only available at coarser spatial resolution.

We resampled all environmental layers to 100 m pixel size where necessary, as this cell size is similar in area to the smallest of the two species’ home ranges (hutia: 26,590 m^2^; solenodon: 159,750 m^2^; Kennerley, Nicoll, Butler, et al., 2019). We calculated home range summary data centered in each cell (continuous predictors: mean and standard deviation; categorical predictors: count and majority) for use in solenodon analyses to enable use of the same grid for both species.

### 2.3 Species distribution modeling

Maximum entropy modeling, implemented in maxent version 3.4.1 (Phillips, Anderson, & Schapire, 2006), was selected over other SDM approaches for its superior accuracy when presence‐only sample sizes are <100 (Hernandez, Graham, Master, & Albert, 2006; Pearson, Raxworthy, Nakamura, & Peterson, 2007; van Proosdij, Sosef, Wieringa, & Raes, 2016).

We assessed the predictive accuracy of the models with area under the curve (AUC), estimated using 25% of the presence data retained for use as “test” data for SDMs created with the remaining “training” data. As maxent restricts the features it tests for sample sizes containing <80 presence records, features were manually limited in all models for each species to those selected by maxent for the smallest dataset for that species, to reduce incongruity between models due to variation in sample size. Default regularization parameters were used throughout. We assessed relative contribution of predictors to the model using the “heuristic estimate” (%) calculated by maxent. If the permutation importance of a predictor (the % decrease in AUC resulting from removal of predictor) was 0%, it was removed from the model, and predictors that contributed the least were removed in a stepwise fashion while ensuring AUC was >0.80 until the most parsimonious number of predictors (the final predictor set) was reached (van Gils, Conti, Ciaschetti, & Westinga, 2012), to reduce the risk of overfitting (Anderson & Gonzalez, 2011). When a selected predictor was categorical (geology and land cover classifications), each category was considered a positive predictor when associated with >0.5 probability of presence. We generated average SDMs based on the final predictor set with the number of random‐seed bootstrapped runs set to 10, with model robustness assessed using the standard deviations of these 10 duplicate runs. We thinned all presence data using the “spThin” package in R (Aiello‐Lammens, Boria, Radosavljevic, Vilela, & Anderson, 2015), such that the minimum distance between records was the diameter of a circular home range for each species, to reduce the likelihood of spatial autocorrelation in the data. This process resulted in the final survey dataset being reduced to 262 solenodon and 187 hutia presence records, and the final opportunistic dataset being reduced to 131 solenodon and 46 hutia presence records.

We produced three different SDMs for each species: (1) using opportunistic data, with a starting model that excluded forest cover, land cover type, and distance to road because many records substantially pre‐date current‐day patterns of land use (the “opportunistic model”); (2) using 2010–2012 survey data, with a starting model that incorporated all predictors (the “total survey model”); and (3) using 2010–2012 survey data, with a starting model that also excluded forest cover, land cover type, and distance to road to allow further comparison with the abiotic‐only opportunistic data SDM (the “reduced survey model”). Models produced using 2010–2012 survey data incorporated a bias file to describe spatial variation in survey effort, as systematic survey effort was restricted to spatially discrete protected areas.

We compared models in two ways. First, we used AUC to measure the accuracy of a given model in predicting presence records in the full dataset collected using the alternative data collection method (i.e., survey data to test opportunistic model, and opportunistic data to test both survey models). Second, we compared different models for each species using three metrics of similarity in ENMtools (Warren, Glor, & Turelli, 2010): Pearson correlation values based on cell‐by‐cell comparison, Schoener's D (Schoener, 1970), and Hellinger distance (I; Warren, Glor, & Turelli, 2008). Metrics range from 0 (species have completely discordant distribution models) to 1 (species have identical distribution models) (Warren et al., 2010).

## 3 RESULTS

### 3.1 Solenodon models

Heuristic contributions of environmental variables and AUC for all final solenodon models are given in Table 2.

**TABLE 2:** Heuristic contributions of environmental variables (%) and AUC for final solenodon and hutia models. Key: *, home range mean value rather than grid cell value; **, home range majority type (i.e., final model included majority land cover within an equivalent solenodon home range centered in that grid cell, rather than habitat type within grid cell; see Methods for further details).

Final model	Elevation	Slope	Aspect (cos)	Aspect (sin)	% forest	Dist. to road	Geology	Land cover	AUC (*SD*)
Solenodon									
Opportunistic	20.0	19.4	7.4	7.3	*N*/A	*N*/A	46.0	*N*/A	0.877 (0.016)
Total survey	16.3*	—	—	—	19.4	—	31.7	32.7**	0.925 (0.007)
Reduced survey	29.7	—	–7.8	–12.0	*N*/A	*N*/A	50.5	*N*/A	0.893 (0.012)
Hutia									
Opportunistic	42.6	—	–9.3	–9.1	*N*/A	*N*/A	39.0	*N*/A	0.885 (0.024)
Total survey	19.8	—	—	—	15.0	—	27.9	37.3	0.895 (0.015)
Reduced survey	40.7	12.5	—	—	*N*/A	*N*/A	46.8	*N*/A	0.847 (0.021)

In the final opportunistic model, probability of presence was >0.5 in marble (0.73 ± 0.09), limestone (0.60 ± 0.01), and marl (0.55 ± 0.06) (*geology type*). Probability of presence (*elevation*) had two peaks in >0.5 probability (below 500 m and ~1,200 m). There was a probability of presence only where slope was <10.

In the final total survey model, probability of presence was >0.5 in woody agriculture (cacao/coffee) (0.60 ± 0.04), semi‐deciduous woodland (0.62 ± 0.02), and evergreen cloud forest (0.57 ± 0.03) (*land cover type*), and in Quaternary sediment (reef limestone, sand, conglomerate) (0.65 ± 0.05) and limestone (0.58 ± 0.01) (*geology type*). Probability of presence increased with tree cover, with a minimum of 50% cover required for probability = 0.5, increasing linearly to 100% tree cover. Probability of presence (*elevation*) had two peaks in >0.5 probability (below 500 m, and ~1,200 m).

In the final reduced survey model, probability of presence was >0.5 in Quaternary sediment (reef limestone, sand, conglomerate) (0.61 ± 0.12) and limestone (0.58 ± 0.02) (*geology type*). Probability of presence (*elevation*) had two peaks in > 0.5 probability (below 900 m and ~1,900 m).

### 3.2 Hutia models

Heuristic contributions of environmental variables and AUC for all final hutia models are given in Table 2.

In the final opportunistic model, probability of presence declined rapidly with elevation, with <0.5 probability above 49 m. Probability of presence was >0.5 in marsh substrate (0.65 ± 0.09) and limestone (0.51 ± 0.02) (*geology type*).

In the final total survey model, probability of presence was >0.5 in woody agriculture (cacao/coffee) (0.74 ± 0.03), evergreen cloud forest (0.64 ± 0.10) and mangrove (0.54 ± 0.04) (*land cover type*), and limestone (0.58 ± 0.03) (*geology type*). There was greater probability of presence at low elevations, with no cells with >0.5 probability above 125 m. Probability of presence increased with tree cover, becoming 0.5 at 40% cover and increasing rapidly between 90% and 100% cover.

In the final reduced survey model, probability of presence was >0.5 only in limestone (0.59 ± 0.03) (*geology type*) and where slope was <22˚. There was greater probability of presence at low elevations, with >0.5 probability occurring only below 100 m.

### 3.3 Model comparisons

Models based on one data collection method predicted the location of presence records collected via the alternative method with better than random accuracy (>0.5) for both species, but too poorly to be considered “good” models (<0.8), with all models achieving a predictive accuracy of 0.63–0.77. For hutia, there was little difference in the ability of the survey model (AUC; full = 0.701 ± 0.038, reduced = 0.689 ± 0.037) and the opportunistic model (0.684 ± 0.014) to predict the other data type. For solenodon, however, the opportunistic model was better at predicting survey data (0.776 ± 0.013) than the survey models predicted opportunistic data (full = 0.654 ± 0.024, reduced = 0.631 ± 0.023).

**FIGURE 4:**
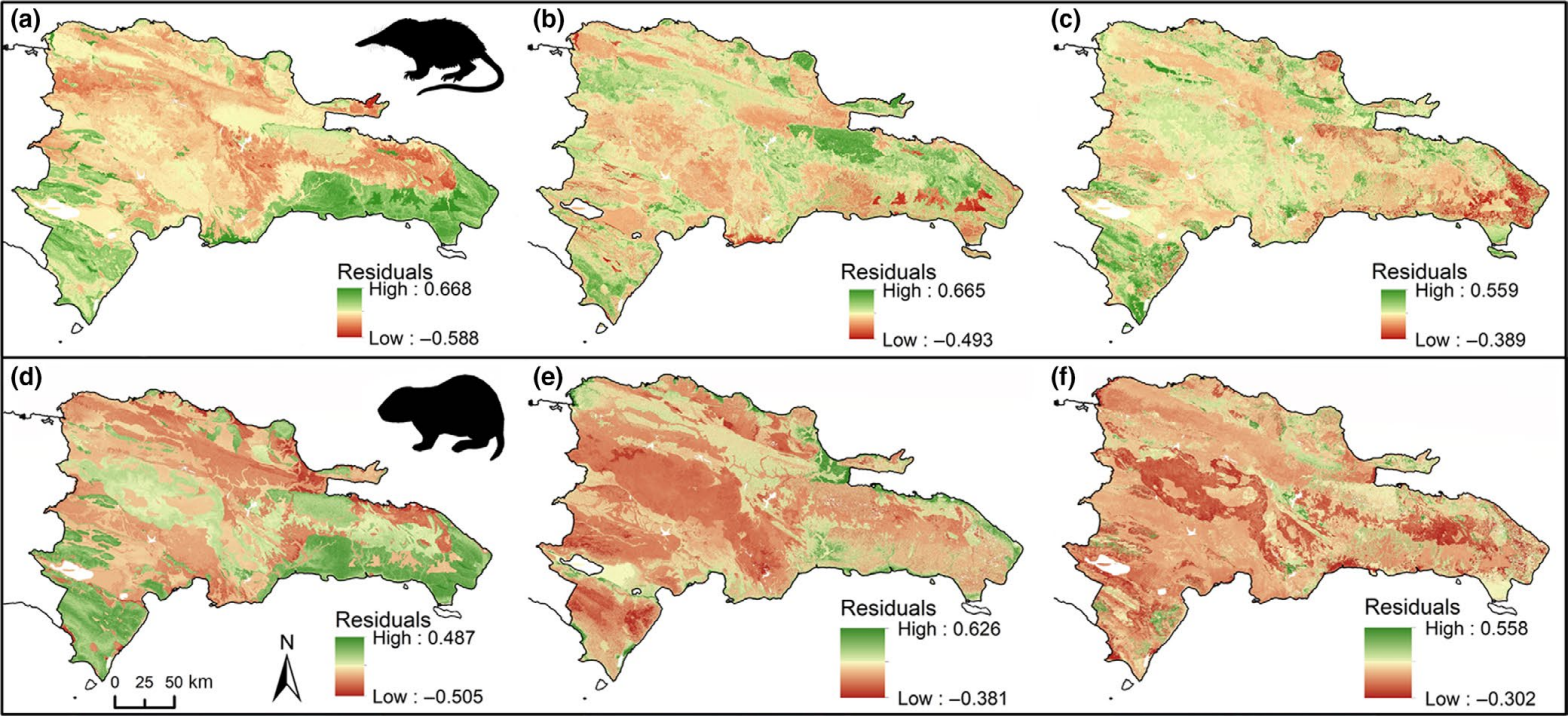
Correlation residuals for (a–c) Hispaniolan solenodon (*Solenodon paradoxus*) and (d–f) Hispaniolan hutia (*Plagiodontia aedium*) of: (a and d) reduced survey model against opportunistic model; (b and e) opportunistic model against total survey model; and (c and f) total survey model against reduced survey model

For both species, cell values showed moderately positive correlation between opportunistic and total survey models (correlation coefficients: solenodon = 0.504; hutia = 0.503), slightly greater correlation between opportunistic and reduced survey models (solenodon = 0.543; hutia = 0.504), and close correlation between total survey and reduced survey models (solenodon = 0.905; hutia = 0.874) (Table 3), with maps of the residuals from these correlations showing patterns of spatial congruence between models (Figure 4). Measures of niche overlap between all model pairwise comparisons were high, with solenodon values slightly higher than hutia values (value ranges: D = 0.669–0.864, I = 0.908–0.983; Table 3).

**TABLE 3:** Pairwise comparisons of species distribution models developed for Hispaniolan solenodon (*Solenodon paradoxus*) and Hispaniolan hutia (*Plagiodontia aedium*) using three different sets of occurrence data, compared using Pearson correlation values, Schoener's D, and Hellinger distance (I)

Species	Model pairwise comparison	Pearson's correlation	D	I
Solenodon	Opportunistic—total survey	0.504	0.721	0.933
	Total survey—reduced survey	0.905	0.864	0.983
	Opportunistic—reduced survey	0.543	0.710	0.928
Hutia	Opportunistic—total survey	0.503	0.669	0.908
	Total survey—reduced survey	0.874	0.827	0.972
	Opportunistic—reduced survey	0.504	0.678	0.917

## 4 DISCUSSION

Our study establishes an important new spatially explicit conservation baseline for understanding the country‐level distribution of two poorly understood but global‐priority threatened land mammals, the Hispaniolan solenodon and Hispaniolan hutia, across the Dominican Republic. This baseline provides the first comparative assessment of predicted spatial distributions and priority conservation landscapes for these species, and our species distribution modeling approach permits wider evaluation of the congruence and relative information content of the two different major types of spatial data that are available for research and management in many other threatened taxa.

Although available ecological data on Hispaniolan mammals are limited and sometimes contradictory (Kennerley, Nicoll, Butler, et al., 2019), environmental variables associated with increased likelihood of solenodon or hutia presence in our SDMs are broadly consistent with species‐specific geomorphological and habitat requirements reported in previous descriptive or fine‐scale studies, providing support for the likely overall accuracy of our models in predicting species’ ecological niches. Our final solenodon SDMs include increased probability of presence on limestone and other carbonate‐rich sedimentary or metamorphic bedrocks; at both moderate elevations (below 500 m or 900m) and high elevations (~1,200 m or ~1,900 m); in areas with low slope (<10°); in primary forest types (semideciduous woodland and evergreen cloud forest) and woody agriculture; and in areas of high tree cover. Hispaniolan solenodons have often been considered dependent on stony forest (Allen, 1942; Miller, 1929). Qualitative country‐wide assessment of solenodon occurrence across the Dominican Republic by Ottenwalder (1999) suggested they typically occur in steep hilly or mountainous terrain and coastal lowlands, most frequently at moderate elevations (below 800 m) but up to 1,500 m and possibly to 2,000 m, and with limestone (karst and reef formations) the dominant rock type in most locations, although they also occur on igneous and metamorphic rocks at high elevations. The remnant solenodon population in Haiti shows a similar distributional pattern (Turvey, Meredith, & Scofield, 2008; Woods & Ottenwalder, 1992). Ottenwalder (1999) reported solenodons from a range of subtropical broadleaf forest types on shallow soils, with old mature primary forest considered optimal habitat although they might persist at least temporarily in disturbed secondary forest; however, a recent multiyear radiotelemetry research program demonstrated that solenodons regularly occur in cash‐crop plantations, subsistence agriculture and pasture, and closed‐canopy forest (Kennerley, Nicoll, Butler, et al., 2019). Previous analysis with multimodel inference of the systematic survey dataset used in this study has shown that lower elevation, increased surrounding tree cover, and canopy closure are all associated with increased probability of detecting solenodons (Kennerley, Nicoll, Young, et al., 2019).

Our final hutia SDMs include increased probability of presence at much lower elevations than solenodons (>0.5 probability below 49 m, 100 m, or 125 m in different models); on limestone and on marsh; in primary forest types (evergreen cloud forest, mangrove) and modified landscapes retaining canopy cover (woody agriculture); and in areas of high tree cover. Previous studies have indicated that although hutias are dietary generalists (Woods & Ottenwalder, 1992) and occur in dry and humid broadleaf forest types, in the Dominican Republic they are dependent upon limestone substrate and intact forest containing large trees to provide cavities for denning, and are apparently absent from areas of volcanic rock (Sullivan, 1983). Radiotelemetry has demonstrated that unlike solenodons, hutias are almost exclusively restricted to closed‐canopy forest in the southwestern Dominican Republic (Kennerley, Nicoll, Butler, et al., 2019), and increased canopy closure and older‐growth forest, as well as increased rock substrate (providing more den sites), are associated with increased probability of detecting hutias across the Dominican Republic in multimodel inference using our systematic survey data (Kennerley, Nicoll, Young, et al., 2019). Conversely, hutias have also been considered locally more abundant than solenodons in modified landscapes in Haiti, and are potentially better able to tolerate disturbance (Woods, 1981). Our predicted higher probability of hutia presence in mangroves, on marsh substrate, and at low elevations based on recent survey data is consistent with independent historical observations from coastal swamp forest and mangrove (Miller, 1927; Sullivan, 1983). Hispaniolan hutias might therefore be ecologically comparable to Cuban hutia species that are either mangrove‐dependent (*Mesocapromys* spp.) or have different populations occurring in mangroves and inland forest (*Capromys pilorides*) (Borroto‐Páez & Mancina, 2011; Luther & Greenberg, 2009), and this habitat type might be important for long‐term persistence of the species.

All of our SDMs predict that both solenodons and hutias are likely to occur over a broadly congruent and relatively large area of the Dominican Republic, including low‐elevation regions across the eastern part of the country, in promontories along the northern coast, and in the southern Sierra de Neiba, Sierra de Bahoruco, and Jaragua Peninsula at a range of elevations. These wide predicted distributions and ecological tolerances, coupled with the generalist diets recorded for both species (Ottenwalder, 1991, 1999; Woods & Ottenwalder, 1992), may help to explain why solenodons and hutias were able to survive the severe postglacial extinction event that eliminated most of Hispaniola's endemic land mammal species (Turvey, 2009), some of which are known to have had more restricted intraisland distributions (Cooke, Rosenberger, & Turvey, 2011; Woods, 1989). Conversely, both solenodons and hutias are predicted to be largely absent from much of the central and northern Dominican Republic, including most of the mountainous Cordillera Central and Cordillera Septentrional. Although our SDMs suggest that solenodons have increased probability of presence at high and moderate elevations, the Cordillera Central might be less ecologically suitable for both species because its bedrock is largely granitoid and volcanic (Iturralde‐Vinent & MacPhee, 1999; Lapierre et al., 1997; Mann, Draper, & Lewis, 1991). We also predict low habitat suitability for both species in the Neiba Valley or Enriquillo Graben, a prominent geological depression in southern Hispaniola, which acts as the boundary between the distributions of allopatric northern and southern solenodon and hutia subspecies (Brace et al., 2012; Ottenwalder, 2001; Turvey et al., 2015, 2016). This landscape feature was at least periodically inundated to form a narrow seaway until the late Pleistocene (Graham, 2003; Maurrasse, Pierre‐Louis, & Rigaud, 1982), and our SDMs indicate the region remains a barrier to gene flow in native land mammals due to current‐day habitat unsuitability.

The broad predicted country‐level distributions for both solenodons and hutias, and the general congruence in all predicted distributions, suggest that country‐level spatial conservation prioritization through the Dominican Republic's extensive existing protected area network should cover key habitats for both species. However, SDMs are only able to generate predictions about where species are expected to occur based on available environmental parameters (Franklin, 2009; Guisan et al., 2013), and predicted habitat suitability does not necessarily indicate continued survival (Burgio, Carlson, & Tingley, 2017; Chatterjee, Tse, & Turvey, 2012; Chen et al., 2018). Although our SDMs indicate suitable environmental conditions are still present across large areas of the Dominican Republic, and local hunting of native mammals is thought to have ceased, solenodon and hutia populations might still be reduced or absent in areas of good‐quality habitat due to competition or predation by invasive mammals (Turvey et al., 2014). Furthermore, land cover and tree cover are included within final total survey models for both solenodons and hutias, with probability of presence increasing with tree cover, but forest loss in the Dominican Republic is estimated at >11% per year (higher than regional averages for the Neotropics) and is accelerating, even within many protected areas (Lloyd & León, 2019; Pasachnik, Carreras De León, & León, 2016; Sangermano et al., 2015), and with tourism infrastructural development impacting mangrove ecosystems required by hutias (Meyer‐Arendt, Byrd, & Hamilton, 2013). Our SDMs therefore predict the distribution of current conservation‐priority landscapes for both species, but these landscapes require further fieldwork to investigate continued presence of native mammals, especially for regions with predicted habitat suitability but lacking records (e.g., Sierra de Neiba), combined with targeted spatial management to maintain key habitat integrity into the future.

Correlation between our total survey and reduced survey models is, unsurprisingly, high for both species. However, correlation between survey models and opportunistic models is only moderately positive (Table 3) and exhibits spatial variation in correlation for both species (Figure 4), with incomplete congruence in spatial distribution of predicted suitable habitat between SDM data types (Figures 2 and 3). This variation could reflect a series of potential differences between opportunistic and survey data, associated with both data quality and data quantity. We consider it unlikely that reduced correlation in our models is associated with variation in either spatial error in record precision (i.e., locational error) or differences in sample size (between different data types or between species). maxent has been shown to be robust to both of these sources of variability, and although model accuracy decreases and variability increases across species and between models with decreasing sample size, maxent exhibits the best predictive power across a range of SDM algorithms and generates similar overall distributional patterns even at much smaller sample sizes to those used in this study (Graham et al., 2008; Papes & Gaubert, 2007; Wisz et al., 2008). Indeed, although historical data can include mixed‐scale records and can generate greater predicted areas in SDMs resulting from resolution mismatch between coarser species records and environmental predictors (Reside, Watson, VanDerWal, & Kutt, 2011), our models show similar model performance (AUC) scores for both opportunistic and survey datasets. Both species included in this study are also morphologically distinctive, reducing the risk of model error associated with misidentification in occurrence records (Aubry, Raley, & McKelvey, 2017; Frey, Lewis, Guy, & Stuart, 2013; Lozier, Aniello, & Hickerson, 2009).

Differences in systematic versus opportunistic model fit and associated distributional patterns between solenodons and hutias may partly reflect species‐specific differences in predictive power of biotic and human impact parameters, which were not incorporated within our opportunistic SDMs. However, whereas previous studies of SDM performance have tended to focus on the effect of variable availability and precision of locality records (Pearson et al., 2007; Reside et al., 2011), we consider it more likely that our model predictions based on different types of distribution data vary due to incompleteness and bias in spatial sampling associated with both data types, which can generate errors in commission and omission that can be hard to identify or quantify (Boakes et al., 2010). Our opportunistic data might be affected by survey bias toward more easily accessible sites (e.g., at lower elevations) and/or preferential resampling by museum collectors of areas with known records, as suggested by previous researchers (Ottenwalder, 1999). In addition, several landscapes in the Dominican Republic with historical mammal records have experienced extensive recent habitat modification, for example through agricultural conversion and mining (Ottenwalder, 1999), in particular in historical lowland marsh/mangrove hutia sites (Sullivan, 1983), making such sites unlikely to be identified as suitable habitat in SDMs that include recent biotic or human impact parameters. Conversely, although our systematic data were obtained from multiple protected areas distributed across the Dominican Republic and represent intensive survey effort across a broad range of habitats, vegetation types, and topographic/climatic variables, these still represent a discrete subset of the country's landscapes, and greater survey effort was invested in the Sierra de Bahoruco given its high elevational and habitat variation. In contrast, opportunistic data could theoretically derive from across the entire country. This inevitable spatial variation in systematic survey effort is associated with increased predicted habitat suitability in systematic SDMs for areas around our survey landscapes, for example around Sierra de Bahoruco and Jaragua NPs in southern Dominican Republic, and across the extensive region of Quaternary carbonate sediments in eastern Dominican Republic, which includes Del Este NP and Punta Cana Ecological Reserve (Díaz de Neira et al., 2017). However, we note that historical mammal records for the southern Dominican Republic are relatively limited, so that variation between models in predicting species occurrence in this region also likely represents an omission error in our opportunistic data.

We did, however, find differences in the ability of models derived from one data collection method to predict the location of presence records collected via the alternative method. For solenodons, the opportunistic model was better at predicting survey data compared with the predictive ability of survey models for opportunistic data. This difference is likely to be associated with the different time periods represented by these different datasets. The opportunistic dataset includes historical records that might have been collected from areas within the species’ former range, but from which it has been subsequently extirpated. As a result, the opportunistic model might represent the distribution of suitable solenodon habitat in the absence of current‐day threats, whereas the survey models, which are based only on recently collected data, instead represent the subset of suitable habitat occupied today. Using presence records from different time periods might therefore provide unrealistically broad SDM predictions if human disturbance has increased over time. Conversely, the opportunistic model was not a better predictor in the hutia analyses, possibly due to differences in habitat preferences between the two species. As hutias are forest specialists, both modern and historical datasets will be likely to identify forest (or associated geology) as suitable habitat. Conversely, solenodons occur across a wider range of habitat types, and so range changes over time might be associated with reduction or exclusion from specific habitat types that would therefore no longer be represented in SDM predictions. This hypothesis is particularly likely given that human impacts in the Caribbean and elsewhere are spatially and environmentally nonuniform, and have affected specific habitat types and landscapes more severely than others (Ottenwalder, 1999; Sullivan, 1983).

Our comparative investigation into the relative information content and predictive power of different types of spatial occurrence data indicates that models derived from different data types can provide different predictions about habitat suitability and conservation‐priority landscapes for threatened species, with discrepancies between models likely reflecting unevenness in spatial data coverage associated with both data types. Explicit awareness must therefore be made of potential incompleteness and bias in models derived from both opportunistic and systematic datasets, with neither data type being inherently more accurate at predicting “true” species distributions. Given this uncertainty, we suggest that our model outputs could be used to define key conservation‐priority landscapes for Hispaniolan mammals as areas with congruent high predicted suitability in both opportunistic and survey models, and particularly those areas with high predicted suitability across all models for both species. Large‐scale surveys of threatened species often require extensive investment in funding, resources, time, and training, and collection of our Hispaniolan mammal systematic survey dataset represented an exhaustive multi‐year effort (Kennerley, Nicoll, Young, et al., 2019). It is therefore necessary to evaluate the resources required to gather sufficient evidence and establish spatial conservation baselines using either literature reviews of anecdotal data or rigorous large‐scale data collection efforts (Cook, Pullin, Sutherland, & Stewart, 2017), within the context of existing data quality and availability for poorly studied species, cost‐effectiveness of research approaches, and feasibility of accurate and representative field‐based data collection that can meaningfully inform future conservation.

## CONFLICT OF INTEREST

None declared.

## AUTHOR CONTRIBUTIONS

**Samuel T. Turvey:** Conceptualization (equal); Data curation (equal); Funding acquisition (supporting); Investigation (equal); Methodology (equal); Project administration (equal); Visualization (equal); Writing‐original draft (lead); Writing‐review & editing (lead). **Rosalind J. Kennerley:** Formal analysis (equal); Investigation (equal); Methodology (equal); Resources (equal); Writing‐original draft (supporting); Writing‐review & editing (supporting). **Michael A. Hudson:** Formal analysis (equal); Investigation (equal); Methodology (equal); Writing‐original draft (supporting); Writing‐review & editing (supporting). **Jose M. Nuñez‐Miño:** Conceptualization (equal); Data curation (equal); Investigation (lead); Methodology (supporting); Project administration (equal); Writing‐original draft (supporting); Writing‐review & editing (supporting). **Richard P. Young:** Conceptualization (equal); Data curation (supporting); Funding acquisition (lead); Investigation (supporting); Methodology (supporting); Project administration (equal); Supervision (supporting); Writing‐original draft (supporting); Writing‐review & editing (supporting).

## Data Availability

All supporting data are available online at University College London's Research Data Repository (https://doi.org/10.5522/04/11993388.v1, https://doi.org/10.5522/04/11993424.v1).
